# Marine Capture Fisheries from Western Indian Ocean: An Excellent Source of Proteins and Essential Amino Acids

**DOI:** 10.3390/foods12051015

**Published:** 2023-02-27

**Authors:** Ida-Johanne Jensen, Nathalie Bodin, Rodney Govinden, Edel Oddny Elvevoll

**Affiliations:** 1 Norwegian College of Fishery Science, Faculty of Biosciences, Fisheries, and Economics, UiT-The Arctic University of Norway, N-9037 Tromsø, Norway; 2 Department of Biotechnology and Food Science, Norwegian University of Science and Technology, NTNU, 7491 Trondheim, Norway; 3 Seychelles Fishing Authority (SFA), Fishing Port, Victoria P.O. Box 449, Mahé, Seychelles; 4 Sustainable Ocean Seychelles, BeauBelle, Mahé, Seychelles

**Keywords:** Small Island Developing States (SIDS), pelagic and reef species, essential nutrients, ocean food security, amino acid composition, protein

## Abstract

The Republic of Seychelles is located in Western-Central Indian Ocean, and marine capture fisheries play a key role in the country’s economic and social life in terms of food security, employment, and cultural identity. The Seychellois are among the highest per capita fish-consuming people in the world, with a high reliance on fish for protein. However, the diet is in transition, moving towards a Western-style diet lower in fish and higher in animal meat and easily available, highly processed foods. The aim of this study was to examine and evaluate the protein content and quality of a wide range of marine species exploited by the Seychelles industrial and artisanal fisheries, as well as to further to assess the contribution of these species to the daily intake recommended by the World Health Organization (WHO). A total of 230 individuals from 33 marine species, including 3 crustaceans, 1 shark, and 29 teleost fish, were collected from the Seychelles waters during 2014–2016. All analyzed species had a high content of high-quality protein, with all indispensable amino acids above the reference value pattern for adults and children. As seafood comprises almost 50% of the consumed animal protein in the Seychelles, it is of particular importance as a source of essential amino acids and associated nutrients, and as such every effort to sustain the consumption of regional seafood should be encouraged.

## 1. Introduction

The significance of global food and nutrition security is anchored in the United Nations Sustainable Development Goals (SDGs) SDG2 “Zero Hunger” and SDG 3 “Good Health and Well-Being” [1]. It is strongly encouraged that an increased food production should come from well-managed ocean resources. Land-based resources are limited, and agricultural food production is one of the major greenhouse gas (GHG) emitters [2,3]. 

Seafood plays an important role in food and nutrition security, particularly in low- and middle-income countries [4]. The nutritional recommendations to eat fish are based on their lipid content and fatty acid composition [5], although seafood is also an important source of vitamins and minerals [6,7] and high-quality proteins [8,9] that are important for human health and disease prevention. Seafood is also recognized as a rich source of taurine [10], considered to have a positive impact on cardiovascular diseases [11,12]. Seafood may also be a source of toxic heavy metals such as mercury, arsenic, lead, and cadmium [6,13,14], as well as persistent organic pollutants such as dioxins and dioxin-like polychlorinated biphenyls (PCBs) [15,16,17,18]. However, public agencies in Europe have reviewed available evidence through 2021 and concluded that the possible adverse effects of mercury, dioxin, and dioxin-like PCB exposure are offset by the benefits of seafood consumption on cardio-metabolic diseases in general [16,17] and that seafood consumption during pregnancy is likely to benefit the neurocognitive development of children [15]. Additionally, a relatively recent review conducted by Hibbeln et al. [19] concluded with moderate and consistent evidence that seafood consumption during pregnancy and childhood had beneficial associations with neurocognitive outcomes. Since 1986, an ongoing research project in the Seychelles (Seychelles Child Development Study, SCDS; https://www.urmc.rochester.edu/labs/seychelles.aspx, accessed on 1 January 2023) has been examining associations between maternal methylmercury exposure and neurodevelopment in children [20,21,22].

The Republic of Seychelles, one of the 38 United Nations member states of the Small Island Developing States’ group, is located in Western-Central Indian Ocean. It includes a land surface of only 459 km^2^ divided into 115 tropical islands scattered within an Exclusive Economic Zone (EEZ) of 1.3 million square kilometers [23]. The majority of the population resides on three islands of a large submerged mid-oceanic shelf called the Mahé Plateau. Marine capture fisheries play a key role in the country’s economic and social life. In addition to the industrial tuna fisheries being a major pillar of the economy, artisanal fisheries are continuously of great importance to the local population in terms of food security, employment, and cultural identity. Fish is seen not only as a staple food but also as a delicacy in the local Creole cuisine, and the Seychellois are among the highest per capita fish-consuming people in the world, with a high reliance on fish for protein—consuming about 59 kg per year measured as live weight [23], which is equivalent to 48% of the animal protein consumed [24]. Pregnant women and mothers have been reported to consume as many as 12 meals consisting of fish per week [25]. However, the diet in the Seychelles, as elsewhere, is in transition, moving towards a Western-style diet lower in fish and higher in animal meat and easily available, highly processed foods [26]. This has contributed to the increase in the prevalence of obesity (BMI ≥ 30 kg/m^2^) between 1998 and 2004 from 4 to 15% in men and from 23 to 34% in women [27], and it highlights the importance of fish in the diet. 

Adequate protein intake is essential for tissue maintenance and growth, with amino acids being important as building blocks of proteins and as intermediates in various metabolic pathways. The nutritional quality of a protein is dependent on the content of indispensable, also called essential, amino acids, i.e., amino acids that are not synthesized in our body to meet the human requirements. The World Health Organization (WHO) recommends a daily dietary intake of protein of 830 mg protein/kg body weight for healthy adults; an additional 1, 9, and 31 g protein/day for pregnant women in the first, second, and third trimester, respectively; and 910 mg/kg body weight for children, in addition to specific recommendations for each of the indispensable amino acids [28,29]. 

The protein content of marine capture fisheries can significantly vary between species and even within species depending on habitat, region, and season [30]. Access to local and up-to-date food composition data is therefore essential for dietary counselling, clinical nutrition, and improvements in nutrition security and the development of effective food- and nutrition-related policies [31]. To our understanding, the protein contents and quality of different marine capture species from the Seychelles have not been investigated, nor have any data been published.

The objectives of this work were to examine the amino acid composition and to evaluate the protein content and protein quality of a wide range of marine species exploited by Seychelles industrial and artisanal fisheries, as well as further to assess the contribution of these species to the daily intake of proteins and essential amino acids recommended by the WHO. 

## 2. Materials and Methods

### 2.1. Sample Collection and Preparation

**Figure 1:**
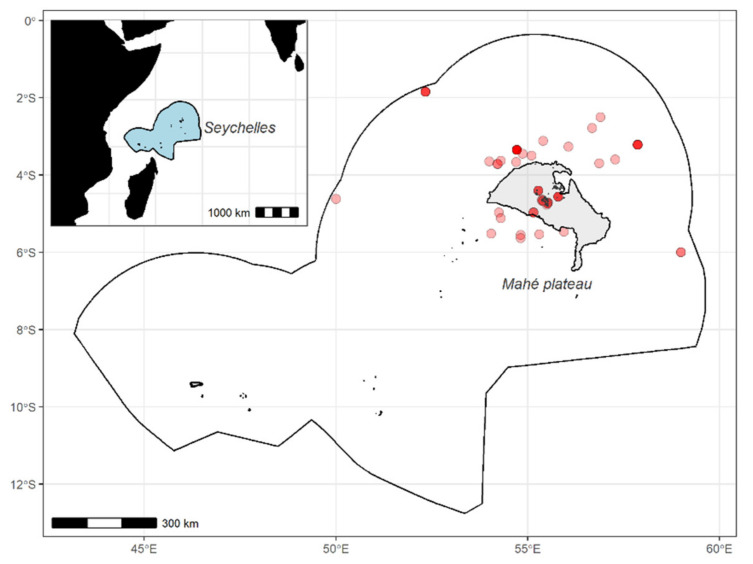
Fishing locations of the 33 marine species on and around the Mahé Plateau, the Seychelles (Western Indian Ocean).

A total of 230 individuals from 33 marine species, including 3 crustaceans, 1 shark, and 29 teleost fish, were collected from Seychelles waters during 2014–2016 (Table 1). Nearshore species were caught on the Mahé Plateau, where most of the artisanal fishing grounds are located [32], and offshore species were caught around the Mahé Plateau within the exclusive economic zone (EEZ) (Figure 1). After their capture, all organisms were measured (cephalothorax length (CL) for crustaceans, lower jaw-fork length (LJFL) for swordfish, and fork length (FL) and total length (TL) for other species) and weighted, and a piece of the edible part was collected from the tail for crustaceans and dorsal muscle for other species before being immediately stored at −80 °C. Samples were then freeze-dried for 72 h and ground to powder before amino acid analyses.

### 2.2. Amino Acid Composition and Protein Content

Amino acid composition was analyzed by dissolving approximately 40 mg of dried samples in 0.7 mL of distilled H_2_O and 0.5 mL of 20 mM norleucine (internal standard), which was then hydrolyzed as previously described [33,34]. Following hydrolysis, 100 µL aliquots of the hydrolysates were evaporated under nitrogen gas until complete dryness and re-dissolved to a suitable concentration in a lithium citrate buffer at pH 2.2. All amino acids were chromatographically analyzed using an ion exchange column followed by ninhydrin post column derivatization on a Biochrom 30 amino acid analyzer (Biochrom Co., Cambridge, UK). Amino acid residues were identified using the A9906 physiological amino acid standard (Sigma Chemical Co., St. Louis, MO, USA), as described previously [35]. The concentrations of 20 amino acids (histidine, his; isoleucine, ile; leucine, leu; lysine, lys; methionine, met; phenylalaline, phe; threonine, thr; valine, val; alanine, ala; b-alanine; b-ala; arginine, arg; asparagine, asn; aspartic acid, asp; cysteine, cys; glutamine, gln; glutamic acid, glu; glycine, gly; hydroxyproline, hyp; proline, pro; serine, ser; tyrosine, tyr; taurine, tau) were converted from dry weight to wet weight by using a mean moisture percentage of 72–81%, depending on species, and expressed in mg per 100 g of raw edible portion (noted mg/100 g). Tryptophan is denatured during acid hydrolysis, while glutamine and asparagine deaminate during acid hydrolysis and were therefore included in the category of glutamate and aspartic acid.

Protein content (g/100 g) was determined as the sum of the individual amino acid residues (the molecular weight of each amino acid after the subtraction of the molecular weight of H_2_O), as recommended by the FAO [36], using norleucine as internal standard.

### 2.3. Statistical Analyses

Statistical analyses were performed using IBM SPSS statistics 27. All samples were measured in duplicate, and the number of individuals analyzed from each marine species is presented in Table 1.

## 3. Results

### 3.1. Protein Content

**Figure 2:**
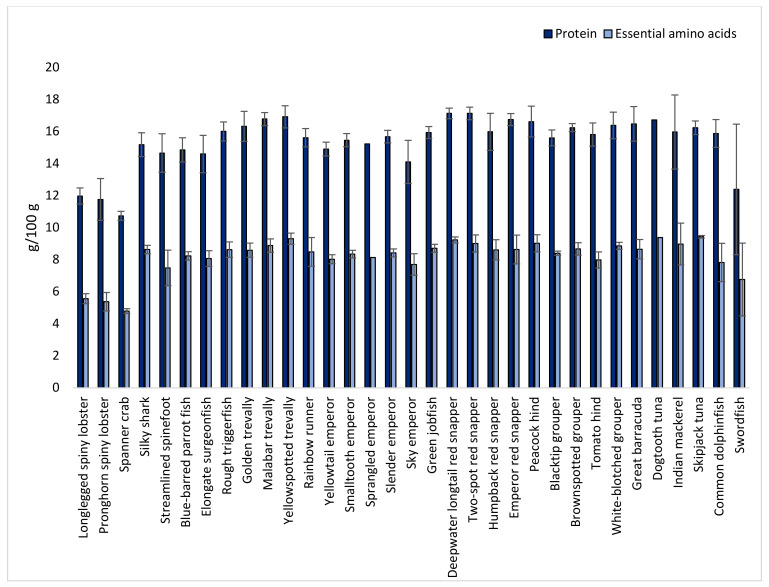
Protein and sum of essential amino acid (threonine, valine, methionine, isoleucine, leucine, phenylalanine, lysine, and histidine; tryptophan is denatured during acid hydrolysis and is thus not included) content (g/100 g) in species caught in the Seychelles waters.

The protein content, calculated as the sum of amino acids minus the molecular weight of water, was relatively constant among all fish species (Figure 2), varying between 13 and 17 g/100 g. Crustaceans had a lower protein content of approximately 11–12 g/100 g. The total amount of essential amino acids (EAAs) constituted half of the protein content for all species (Figure 2).

### 3.2. Distribution of Essential Amino Acids

**Figure 3:**
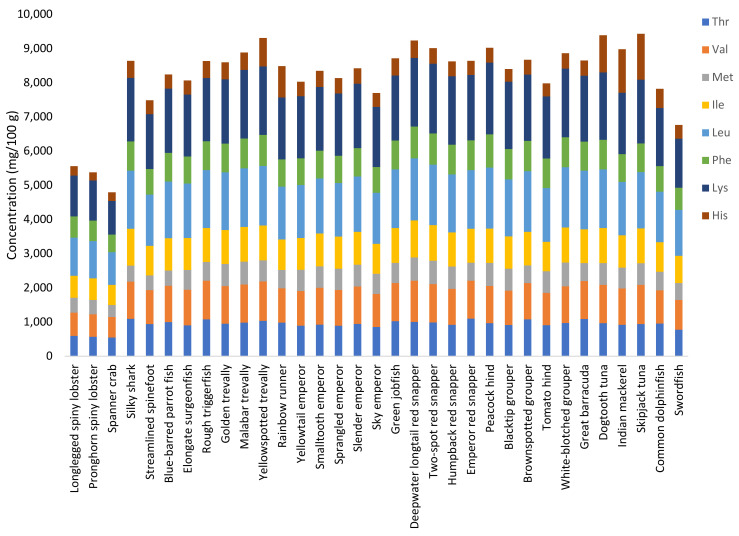
Distribution of essential amino acids (mg/100 g) in species caught in the Seychelles waters. Thr, threonine; Val, valine; Met, methionine; Ile, isoleucine; Leu, leucine; Phe, phenylalanine; Lys, lysine; His, histidine (tryptophan is denatured during acid hydrolysis and is thus not included).

The distribution of EAAs was similar for all fish species (Figure 3), with leucine and the commonly limiting amino acid lysine being the most abundant amino acids (1500–1700 mg/100 g and 1800–2000 mg/100 g, respectively). These two amino acids were also the most abundant in crustaceans, although their contributions were slightly lower than in fish. The concentrations of histidine were highly variable among the 33 studied species (from 250 to 1350 mg/100 g), with the highest relative content being measured in tunas and mackerels. The contents of threonine (776–1093 mg/100 g), valine (867–1091 mg/100 g), methionine (450–697 mg/100 g), isoleucine (799–1071 mg/100 g), and phenylalanine (650–908 mg/100 g) were higher in the fish species compared with the crustaceans (on average 569, 646, 402, 623, and 578 mg/100 g, respectively).

### 3.3. Taurine Concentration

**Figure 4:**
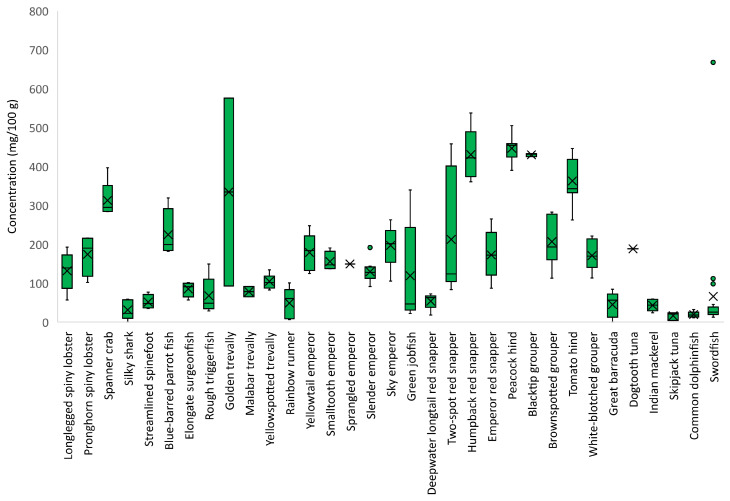
Taurine concentration (mg/100 g) in species caught in the Seychelles waters. Mean values are represented by an ×, median values are represented by −, the boxes cover the interquartile range, and the whiskers represent the minimum and maximum values without outliers. Outliers are plotted as individual points.

The concentration of taurine considerably varied within and among the studied species (Figure 4). Skipjack tuna and common dolphinfish were lowest in taurine (<20 mg/100 g), while humpback red snapper and peacock hind were highest in taurine (440 mg/100 g). 

## 4. Discussion

### 4.1. Protein Content 

The protein content was calculated based on the amount of total amino acids minus the molecular weight of water, as recommended by the FAO [36]. This procedure efficiently hydrolyzes most of the peptide bonds while also reducing some amino acids. Tryptophan is denatured during acid hydrolysis, while glutamine and asparagine deaminate during acid hydrolysis and were therefore included in the category of glutamate and aspartic acid [37]. This may have resulted in a potential underestimation of the actual protein content and a lower protein content compared with that measured with the commonly used Kjeldahl´s method [33]. The protein content was similarly high in all marine species, with the exceptions of spanner crab and lobsters that showed slightly lower protein contents. 

### 4.2. Contribution to Daily Recommended Intake

**Figure 5:**
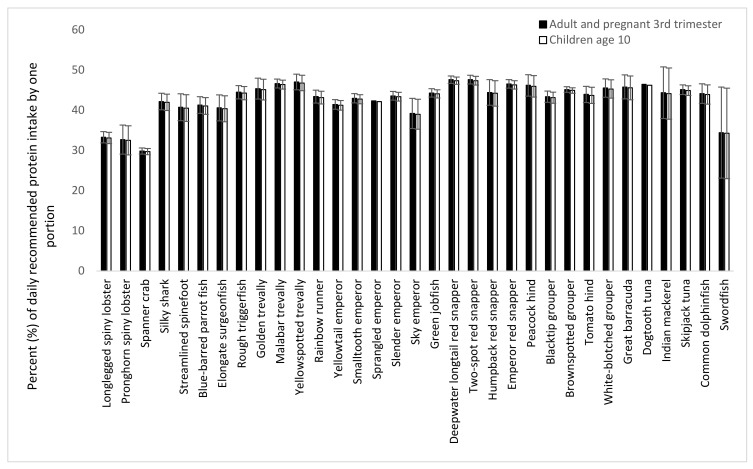
Percent of recommended dietary intake (RDI) of protein for an adult (65 kg), a pregnant woman in the 3rd trimester (65 kg), or for a 10-year-old child (30 kg), that is covered by one portion of 150 or 75 g, respectively. Recommended dietary intake values from Codex [29] and the WHO [28] are used.

The Codex nutritional reference values for protein are based on the best available scientific knowledge of the daily amount needed for good health (830 mg protein/kg body weight for adults and 910 mg kg body weight for children). Based on these reference values and considering a portion size of 150 g for adults and 75 g for children, the contributions of one portion of each capture fishery species to the recommended dietary intake (RDI) for a 65 kg adult person, a 65 kg pregnant woman in the third trimester, and a 10-year-old child (average body weight of 30 kg) were estimated (Figure 5). One portion of swordfish and crustaceans (spiny lobster and spanner crab) would cover 30% of the adult, pregnant woman, and child RDIs. All other species would contribute 40–45% of these RDIs. 

**Figure 6:**
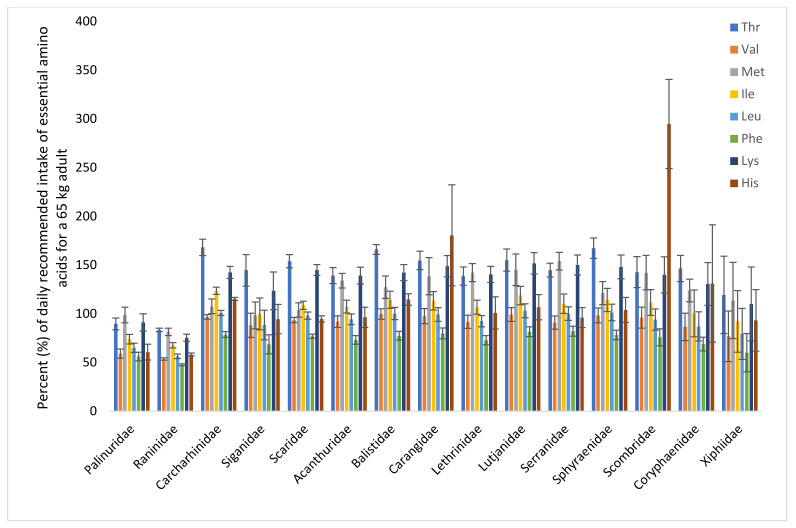
Percent of recommended dietary intake of the essential amino acids for a 65 kg person by a portion of 150 g. Recommended dietary allowance values from the WHO [28] are used. Thr, threonine; Val, valine; Met, methionine; Ile, isoleucine; Leu, leucine; Phe, phenylalanine; Lys, lysine; His, histidine; (tryptophan is denatured during acid hydrolysis and is thus not included).

The FAO and WHO have recommended the dietary intake of each of the indispensable amino acids, based on growth and nitrogen balance. The percentage coverage of each of these amino acids for a 65 kg person by a 150 g portion of different species is illustrated in Figure 6. One portion of crustacean from the Palinuridae (spiny lobster) and Raninidae (spanner crab) families covered 50% of phenylalanine; 60–67% of valine, leucine, isoleucine, and histidine; and 90% of threonine, methionine, and lysine. One portion of fish covered approximately 70% of the daily recommended amount of phenylalanine and around or above 100% of the daily recommended amount of the other indispensable amino acids.

### 4.3. Protein Quality

In addition to being building blocks for protein synthesis, each amino acid has its own metabolic pathway. The 20 proteogenic amino acids are classified as non-essential or essential. The nine essential amino acids (threonine, valine, methionine, isoleucine, leucine, phenylalanine, lysine, histidine, and tryptophan) cannot be synthesized in the human body from naturally occurring precursors at a rate needed to meet the metabolic requirements. 

In this work, protein quality was determined based on the amount of essential amino acids. All analyzed fish species were high in lysine and threonine, which are strictly indispensable [1]. One portion of crustacean meat or fish filet was found to significantly contribute to the daily requirements of the indispensable amino acids, and one portion of fish filet was found to meet the requirements of threonine, methionine, and lysine. However, it is important to mention that all samples were analyzed raw, and several factors may influence amino acid contents during processing and household preparations such as boiling, baking, frying, and smoking [38,39], which may affect the amino acid contribution to the diet. These values thus indicate the amount available in pre-processed food and not the exact actually absorbed amount. The chemical score of amino acids used to assess the amount of limiting amino acids, can be used to determine if a diet meets the required amount of indispensable amino acids. The chemical score equals the ratio between each indispensable amino acid in the food protein and the corresponding amino acid in a reference protein proposed by the FAO/WHO. The protein-digestibility-corrected amino acid score can thereafter be calculated as the amino acid score multiplied by the true digestibility in humans [40]. Proteins of animal source normally have a chemical score of 1.0, while the scores of cereal proteins normally range from 0.4 to 0.6. All species analyzed in this work had a high protein quality, with the contents of all indispensable amino acids above the reference-scoring pattern for adults [40] indicating that the protein quality was superior. As tryptophan was denatured during the acid hydrolysis of the samples, it was not possible to assess whether it is a limiting amino acid.

### 4.4. Taurine

Accumulating evidence supports the idea that an increased dietary intake of taurine, a naturally occurring sulfonic acid, may be beneficial, as it has been documented to attenuate hypertension, suppress atherosclerosis, and exhibit antioxidative and anti-inflammatory properties [41,42,43]. Fish is recognized as a rich source of taurine [44], and urinary taurine may be used as a marker of the level of fish consumption [45]. In this study, taurine content greatly varied not only between species but also within different specimens of a given species. As a free amino acid, taurine is easily lost in handling and preparation, and its content is often found to significantly vary, even within one fillet [38,46]. A stricter control of all parts of the value chain would be necessary to avoid such variation. The levels of taurine measured in this study were within normal ranges compared to seafood in general, but as this is the first report of the levels of taurine for many of these species, comparison is challenging. The highest content was analyzed in the demersal species, humpback red snapper and tomato hind. 

### 4.5. Regional Food and Nutrition Security

Food traditions are important. Food provides nutrients, and changes in lifestyles include nutrition transitions; the decreasing consumption of local foods is often associated with an increase in the consumption of carbohydrate-dense and highly processed foods. Such foods are normally cheaper and excessive in sugar, fat and additives. The high intake of refined food products has led to a worldwide elevated burden of overweight and obesity [47], and Seychellois are not an exception [26]. Malnutrition, excessive caloric consumption, and coexisting micronutrient deficiencies combined with declining activity levels may imply increases in and the earlier onset of lifestyle diseases, and global food systems may be leading to the poorer health of many [48,49,50].

## 5. Conclusions

This study provides detailed information on the concentrations of essential and non-essential amino acids and the protein content and quality of a wide range of tropical capture fishery species from the Seychelles (Western Indian Ocean) caught in both nearshore and offshore waters. The species’ contributions to the recommended daily intake values of indispensable amino acids from the WHO were assessed, and implications for regional food and nutrition security was discussed. 

The captured fish species analyzed in this work had high contents of high-quality protein, with all indispensable amino acids above the reference value pattern for adults and children. Such species with high protein contents of superior quality are perceived as healthy foods. As fish makes up as much as 48% of the consumed animal protein in the Seychelles, it is of particular importance as a source of essential amino acids and associated nutrients such as fatty acids, taurine, vitamins and minerals. Accordingly, every effort to sustain the consumption of regional fish should be encouraged.

## Figures and Tables

**Table 1:** Marine species collected from the Seychelles waters, with associated details. Length (presented as mean ± SD) refers to the mean carapace length for crustaceans, the mean lower jaw-fork length for swordfish, and the mean fork length for other teleost fish and for sharks. N = number of individuals.

Group Family	Scientific Name	English Name	FAO Code	Habitat	Fishing Area	Fishing Year	N	Length	Weight
**Crustacean**									
Palinuridae	*Panulirus longipes*	Longlegged spiny lobster	LOJ	reef-associated	Nearshore	2014	5	8.2 ± 0.7	0.6 ± 0.1
	*Panulirus penicillatus*	Pronghorn spiny lobster	NUP	reef-associated	Nearshore	2014	4	9.3 ± 1.3	0.7 ± 0.2
Raninidae	*Ranina ranina*	Spanner crab	RAQ	reef-associated	Nearshore	2014	5	9.7 ± 1.1	0.3 ± 0.1
**Shark**									
Carcharhinidae	*Carcharhinus falciformis*	Silky shark	FAL	reef-associated	Nearshore	2015	5	79.8 ± 11.4	NA
**Teleost fish**									
Siganidae	*Siganus argenteus*	Streamlined spinefoot	IGA	reef-associated	Nearshore	2015	4	26.0 ± 1.1	0.3 ± 0.0
Scaridae	*Scarus ghobban*	Blue-barred parrotfish	USY	reef-associated	Nearshore	2015	4	29.8 ± 3.5	0.5 ± 0.2
Acanthuridae	*Acanthurus mata*	Elongate surgeonfish	DGW	reef-associated	Nearshore	2016	4	46.8 ± 1.5	2.1 ± 0.1
Balistidae	*Canthidermis maculata*	Rough triggerfish	CNT	reef-associated	Nearshore	2015	5	31.8 ± 3.9	0.7 ± 0.2
Carangidae	*Gnathanodon speciosus*	Golden trevally	GLT	reef-associated	Nearshore	2016	2	66.0 ± 19.8	5.8 ± 4.6
	*Carangoides malabaricus*	Malabar trevally	NGS	reef-associated	Nearshore	2016	2	67.5 ± 6.4	4.6 ± 0.4
	*Carangoides fulvoguttatus*	Yellowspotted trevally	NGU	reef-associated	Nearshore	2016	9	52.4 ± 9.0	2.6 ± 1.2
	*Elagatis bipinnulata*	Rainbow runner	RRU	reef-associated	Nearshore	2015	5	75.1 ± 15.5	NA
Lethrinidae	*Lethrinus crocineus*	Yellowtail emperor	ICZ	reef-associated	Nearshore	2016	5	36.0 ± 5.1	1.0 ± 0.5
	*Lethrinus microdon*	Smalltooth emperor	LEN	reef-associated	Nearshore	2016	4	45.8 ± 2.2	1.5 ± 0.2
	*Lethrinus nebulosus*	Spangled emperor	LHN	reef-associated	Nearshore	2016	1	41	1.2
	*Lethrinus variegatus*	Slender emperor	LHV	reef-associated	Nearshore	2016	10	29.0 ± 1.3	0.4 ± 0.1
	*Lethrinus mahsena*	Sky emperor	LTQ	reef-associated	Nearshore	2016	10	33.7 ± 3.2	0.9 ± 0.2
Lutjanidae	*Aprion virescens*	Green jobfish	AVR	reef-associated	Nearshore	2015	14	52.2 ± 3.3	2.0 ± 0.4
	*Etelis coruscans*	Deepwater longtail red snapper	ETC	reef-associated	Nearshore	2014	10	57.3 ± 11.3	2.8 ± 1.4
	*Lutjanus bohar*	Two-spot red snapper	LJB	reef-associated	Nearshore	2015	15	58.6 ± 15.6	4.4 ± 2.7
	*Lutjanus gibbus*	Humpback red snapper	LJG	reef-associated	Nearshore	2016	9	36.0 ± 3.6	1.0 ± 0.3
	*Lutjanus sebae*	Emperor red snapper	LUB	reef-associated	Nearshore	2014	10	56.8 ± 6.9	4.2 ± 1.5
Serranidae	*Cephalopholis argus*	Peacock hind	CFF	reef-associated	Nearshore	2014	8	30.5 ± 3.3	0.5 ± 0.2
	*Epinephelus fasciatus*	Blacktip grouper	EEA	reef-associated	Nearshore	2016	3	NA	0.4 ± 0.1
	*Epinephelus chlorostigma*	Brownspotted grouper	EFH	reef-associated	Nearshore	2014	9	37.2 ± 2.0	0.7 ± 0.1
	*Cephalopholis sonnerati*	Tomato hind	EFT	reef-associated	Nearshore	2014	9	40.2 ± 4.4	1.1 ± 0.4
	*Epinephelus multinotatus*	White-blotched grouper	EWU	reef-associated	Nearshore	2014	10	64.0 ± 5.6	4.2 ± 1.1
Sphyraenidae	*Sphyraena barracuda*	Great barracuda	GBA	reef-associated	Nearshore	2014	5	106.2 ± 9.0	7.6 ± 2.7
Scombridae	*Gymnosarda unicolor*	Dogtooth tuna	DOT	reef-associated	Offshore	2016	1	93	12.2
	*Rastrelliger kanagurta*	Indian mackerel	RAG	pelagic-neritic	Offshore	2014	10	25.9 ± 0.5	0.3 ± 0.0
	*Katsuwonus pelamis*	Skipjack tuna	SKJ	pelagic-oceanic	Offshore	2014	3	45.4 ± 3.5	2.0 ± 0.4
Coryphaenidae	*Coryphaena hippurus*	Common dolphinfish	DOL	pelagic-neritic	Offshore	2014	10	99.1 ± 7.2	6.4 ± 1.7
Xiphiidae	*Xiphias gladius*	Swordfish	SWO	pelagic-oceanic	Offshore	2014	20	158.9 ± 32.1	NA

## Data Availability

The data are available on request.
